# The interplay between borderline personality disorder and oxytocin: a systematic narrative review on possible contribution and treatment options

**DOI:** 10.3389/fpsyt.2024.1439615

**Published:** 2024-07-23

**Authors:** Ester di Giacomo, Elena Andreini, Jacopo Santambrogio, Alberto Arcara, Massimo Clerici

**Affiliations:** ^1^ Department of Mental Health, Health Care Trust–IRCCS San Gerardo Monza, Monza, Italy; ^2^ School of Medicine and Surgery, University of Milano-Bicocca, Monza, Italy

**Keywords:** borderline personality disorder, oxytocin, empathy, emotional regulation, social cognition, child maltreatment

## Abstract

**Background:**

Borderline personality disorder (BPD) is a complex mental health condition marked by instability in mood, relationships, self-image, and behavior. Individuals with BPD often struggle with intense emotions, impulsivity, and maintaining stable relationships. Oxytocin, known as the "love hormone" or "bonding hormone," plays a crucial role in social bonding, trust, empathy, and emotional regulation and its dysregulation may contribute to BPD difficulties. This systematic review aims to analyze existing literature, examining the intricate interplay and encouraging future research and treatment strategies.

**Methods:**

A systematic search of Literature in PubMed, Embase and Psychinfo, without any language or time restriction, was performed until March 2024 combining thesaurus and free-search indexing terms related to “borderline personality disorder” and “oxytocin”, producing 310 results (77 in PubMed, 166 in Embase and 67 in Psychinfo). Ninety-four full texts were analyzed, and 70 articles were included in qualitative analysis.

**Results:**

Oxytocin may influence attachment styles, parental behaviors, and stress responses, particularly in individuals with a history of childhood trauma. The interaction between oxytocin, genetics, early life experiences, and environmental factors contributes to the complexity of BPD. Genetic variations in the oxytocin receptor gene may influence social and emotional abilities and contribute to the development of psychopathology. Additionally, early adverse experiences, such as childhood maltreatment, can alter oxytocin functioning, impacting social cognition and emotional regulation.However, oxytocin's role in BPD treatment remains uncertain, with some studies suggesting potential benefits for specific symptoms like social threat avoidance, while others indicate adverse effects on nonverbal behavior and mentalizing.

**Conclusion:**

Understanding oxytocin's role in BPD offers insights into potential therapeutic interventions. While oxytocin-based treatments may hold promise for addressing specific symptoms, further research is needed.

## 1 Introduction

Borderline personality disorder (BPD) is a complex mental health condition characterized by pervasive instability in mood, interpersonal relationships, self-image, and behavior. Individuals with BPD often struggle with intense emotions, impulsivity, and difficulties in maintaining stable relationships. Over the years, researchers have explored various biological factors contributing to the development and manifestation of BPD symptoms, and one such area of interest is the role of oxytocin (1).

Oxytocin (OXT), often referred to as the "love hormone" or "bonding hormone," is a neuropeptide that plays a crucial role in social bonding, trust, empathy, and emotional regulation. It is released in response to social interactions, particularly those involving intimacy, nurturing, and positive social affiliations. Given its involvement in regulating emotional responses and social behavior, researchers have hypothesized that dysregulation in the oxytocin system may contribute to the emotional instability and interpersonal difficulties observed in individuals with BPD (2–4).

In recent years, studies investigating the relationship between oxytocin and BPD have yielded intriguing findings, although the exact nature of this relationship remains complex and multifaceted. Some research suggests that individuals with BPD may have alterations in oxytocin levels or sensitivity, which could influence their ability to form and maintain healthy social connections and regulate emotions effectively. Additionally, there is growing evidence that oxytocin-based interventions, such as intranasal oxytocin administration, may have therapeutic potential in alleviating some symptoms of BPD, particularly those related to interpersonal functioning and emotional dysregulation.

However, the relationship between oxytocin and BPD is not without controversy, as studies have yielded inconsistent results, and the precise mechanisms underlying oxytocin's effects on BPD symptoms remain poorly understood. Furthermore, the interplay between oxytocin and other neurobiological, psychological, and environmental factors in the development and progression of BPD requires further exploration.

This systematic review aims to analyze and critically evaluate the existing literature on the relationship between borderline personality disorder and oxytocin. By synthesizing findings from neurobiological, clinical, and therapeutic studies, we seek to enhance our understanding of the complex interplay between oxytocin function and BPD symptomatology, ultimately highlighting potential avenues for future research and therapeutic interventions aimed at improving outcomes for individuals affected by this challenging disorder.

## 2 Methods

Our review was performed in accordance with the Preferred Reporting Item for Systematic Reviews and Meta-analyses (PRISMA) guidelines.

### 2.1 Literature sources and search

A systematic search of Literature was performed in three main databases (PubMed, Embase and PsychInfo) until March 2024. This preliminary exploratory analysis was conducted without any language or time restriction. Search phrases combined thesaurus and free-search indexing terms related to “borderline personality disorder” and “oxytocin”.

### 2.2 Eligibility and exclusion criteria

Studies were considered eligible if analyzing any relationship between borderline personality disorder and oxytocin.

Exclusion criteria were applied to the recruitment and diagnosis of borderline personality disorder which must be through standardized tests. No restriction on OXT detection and biological analysis methods was established.

### 2.3 Data collection process

Three authors (E.d.G., E. A., and A.A.) preliminarily reviewed titles and abstracts of traced articles.The initial screening was followed by the analysis of full texts to check compatibility regarding inclusion and exclusion criteria. Discordances were analyzed and disagreements were resolved by discussion among all the authors.

When reported information was unclear or ambiguous the relevant corresponding author was contacted for clarification.

### 2.4 Data extraction

A standardized form was used to extract data, including information on year of publication, country, setting, characteristics of each study (sample size, age, gender and oxytocin administration or dosage). Two authors (E.A. and A.A.) conducted data extraction independently; extraction sheets for each study were cross-checked for consistency, and any differences were resolved by discussion among the coauthors.

## 3 Results

### 3.1 Literature search and basic information

**Figure 1:**
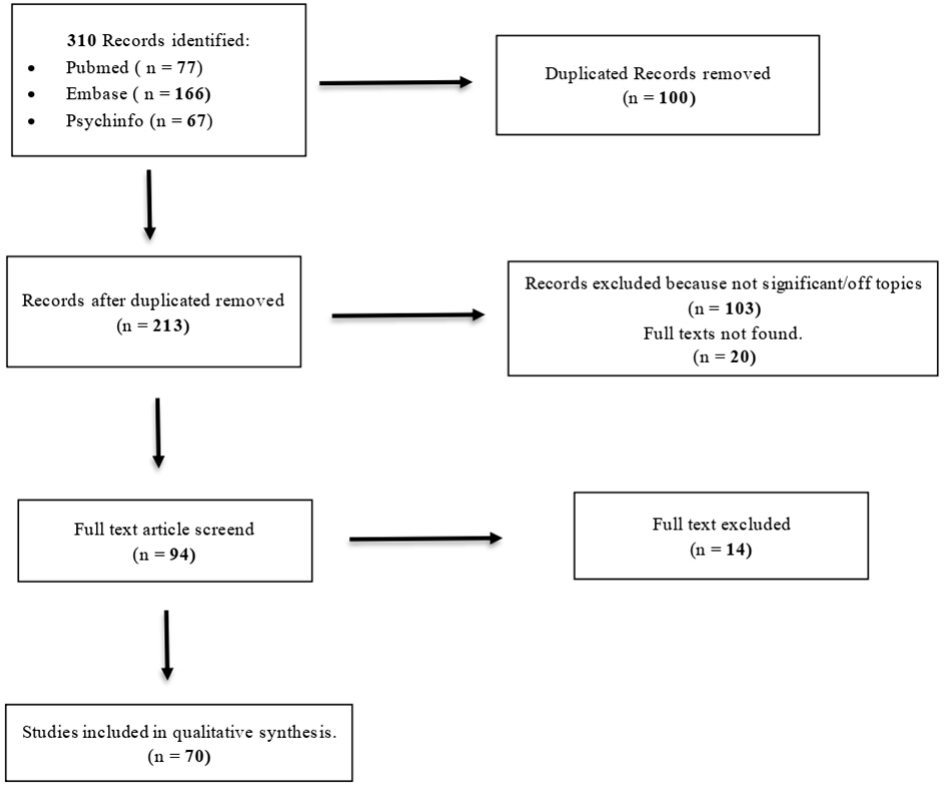
PRISMA flowchart.

The analysis of existing Literature produced 310 results (77 in PubMed, 166 in Embase and 67 in PsychInfo). Ninety-four full texts were analyzed, and 70 articles were included in qualitative analysis. (see **Figure 1**)

**Table 1** provides an overview of the papers included in the qualitative analysis. Papers were sorted in two different categories to facilitate in-depth analysis: correlation between OXT and BPD, and new implications for the BPD treatment.

**Table 1:** Sudy characteristics.

Authors	Year	Title	Theme	Country	Journal
Aboulafia-Brakha, T., Perroud, N., Suchecki, D., Nicastro, R., Dieben, K., & Curtis, L.	2023	Hypomodulation of salivary oxytocin in patients with borderline personality disorder: A naturalistic and experimental pilot study	Correlation	Brazil	*Psychiatry Research Communications*
Back SN , M Schmitz, J Koenig, M Zettl, N Kleindienst, SC Herpertz, K Bertsch	2022	Reduced vagal activity in borderline personality disorder is unaffected by intranasal oxytocin administration, but predicted by the interaction between childhood trauma and attachment insecurity	Treatment	Germany	Journal of Neural Transmission
Bartz J , D Simeon, H Hamilton, S Kim, S Crystal, A Braun, V Vicens, E Hollander	2011	Oxytocin can hinder trust and cooperation in borderline personality disorder	Treatment	USA	Social cognitive and affective neuroscience
Bartz JA, E Hollander	2006	The neuroscience of affiliation: Forging links between basic and clinical research on neuropeptides and social behavior	Correlation	USA	Hormones and behavior
Bertsch K , SC Herpertz	2017	Oxytocin and borderline personality disorder	Correlation /Treatment	Germany	Behavioral pharmacology of neuropeptides: Oxytocin
Bertsch Katja , Ph.D., Matthias Gamer, Ph.D., Brigitte Schmidt, M.D., Ilinca Schmidinger, M.D., Stephan Walther, Ph.D., Thorsten Kästel, M.S., Knut Schnell, M.D., Christian Büchel, M.D., Gregor Domes, Ph.D., and Sabine C. Herpertz, M.D.	2013	Oxytocin and reduction of social threat hypersensitivity in women with borderline personality disorder	Treatment	Germany	*American Journal of Psychiatry*
Bertsch, K., Schmidinger, I., Neumann, I. D., & Herpertz, S. C.	2013	Reduced plasma oxytocin levels in female patients with borderline personality disorder	Correlation	Germany	*Hormones and behavior*
Bomann AC, MB Jørgensen, S Bo, M Nielsen, LB Gede, B Elfving, E Simonsen	2017	The neurobiology of social deficits in female patients with borderline personality disorder: The importance of oxytocin	Correlation	Denmark	Personality and Mental Health
Bonfig J, SC Herpertz, I Schneider	2022	Altered hormonal patterns in borderline personality disorder mother-child interactions	Correlation	Germany	Psychoneuroendocrinology
Brüne M	2016	On the role of oxytocin in borderline personality disorder	Correlation /Treatment	Germany	British Journal of Clinical Psychology
Brüne M , A Ebert, M Kolb, C Tas, MA Edel, P Roser	2013	Oxytocin influences avoidant reactions to social threat in adults with borderline personality disorder	Treatment	Germany	Human Psychopharmacology: Clinical and Experimental
Brüne, M., & Ebert, A.	2012	Does oxytocin have a role in borderline personality disorder?	Treatment	Germany	*International Journal of Neuropsychopharmacology*
Brüne, M., Kolb, M., Ebert, A., Roser, P., & Edel, M. A.	2015	Nonverbal communication of patients with borderline personality disorder during clinical interviews: a double-blind placebo-controlled study using intranasal oxytocin	Treatment	Germany	*The Journal of nervous and mental disease*
Byrd, A. L., Tung, I., Manuck, S. D., Vine, V., Horner, M., Hipwell, A. E., & Stepp, S. D.	2021	An interaction between early threat exposure and the oxytocin receptor in females: Disorder-specific versus general risk for psychopathology and social-emotional mediators	Correlation	USA	*Development and psychopathology*
Carrasco J. L. , E. Buenache, K. S. MacDowell, I. De la Vega, J. M. López-Villatoro, B. Moreno, M. Díaz-Marsá, J. C. Leza	2020	Decreased oxytocin plasma levels and oxytocin receptor expression in borderline personality disorder	Correlation	Spain	*Acta Psychiatrica Scandinavica*
Cataldo, I., Azhari, A., Lepri, B., & Esposito, G.	2017	Oxytocin receptors (OXTR) and early parental care: An interaction that modulates psychiatric disorders	Correlation	Italy	Research in developmental disabilities
Cicchetti, D., Rogosch, F. A., Hecht, K. F., Crick, N. R., & Hetzel, S.	2014	Moderation of maltreatment effects on childhood borderline personality symptoms by gender and oxytocin receptor and FK506 binding protein 5 genes	Correlation	USA	*Development and psychopathology*
Cochran, D. M., Fallon, D., Hill, M., & Frazier, J. A.	2013	The role of oxytocin in psychiatric disorders: A review of biological and therapeutic research findings	Treatment	USA	*Harvard review of psychiatry*
Debbané, M.	2018	Treating borderline personality disorder with oxytocin: An enthusiastic note of caution. Commentary to Servan et al. "The effect of oxytocin in borderline personality disorder.	Treatment	France	*L'encephale*
Diaz-Marsá M , J M López-Villatoro, A De la Torre-Luque, K S MacDowell, A Galvez-Merlin, A Gómez Del Barrio, F Ruiz-Guerrero, L Beato-Fernández, F Polo-Montes, M León-Velasco, D Martín-Hernández, A Carrasco-Diaz, J C Leza, J L Carrasco	2024	Decreased oxytocin plasma levels and oxytocin receptor expression associated with aggressive behavior in aggressive-impulsive disorders	Correlation	Spain	Journal of Psychiatric Research
Domes Gregor , Nicole Ower, Bernadette von Dawans, Franny B. Spengler, Isabel Dziobek, Martin Bohus, Swantje Matthies, Alexandra Philipsen & Markus Heinrichs	2019	Effects of intranasal oxytocin administration on empathy and approach motivation in women with borderline personality disorder: a randomized controlled trial	Treatment	Germany	Translational Psychiatry
Ebert A , MA Edel, P Gilbert, M Brüne	2018	Endogenous oxytocin is associated with the experience of compassion and recalled upbringing in Borderline Personality Disorder	Correlation	Germany	Depression and Anxiety
Ebert, A., Kolb, M., Heller, J., Edel, M. A., Roser, P., & Brüne, M.	2013	Modulation of interpersonal trust in borderline personality disorder by intranasal oxytocin and childhood trauma	Treatment	Germany	*Social neuroscience*
Fineberg SK , DA Ross	2017	Oxytocin and the Social Brain	Correlation	USA	Biological psychiatry
Fisher, Amanda M., et al.	2016	Intranasal oxytocin modulates social cognitive errors in borderline and schizotypal personality disorders	Treatment	UK	Biological Psychiatry
Flasbeck V , D Moser, R Kumsta, M Brüne	2018	The OXTR Single-Nucleotide Polymorphism rs53576 Moderates the Impact of Childhood Maltreatment on Empathy for Social Pain in Female Participants: Evidence for Differential Susceptibility	Correlation	Germany	Frontiers in psychiatry,
Ha, C.	2016	The effects of intranasal oxytocin on social cognitive functioning in adolescents with borderline personality disorder compared to a sample of non-clinical adolescents	Treatment	USA	*Book*
Hammen, C., Bower, J. E., & Cole, S. W.	2015	Oxytocin receptor gene variation and differential susceptibility to family environment in predicting youth borderline symptoms	Correlation	USA	*Journal of personality disorders*
Heinrichs M , B von Dawans, G Domes	2010	Oxytocin, vasopressin, and human social behavior	Correlation /Treatment	USA	Frontiers in neuroendocrinology,
Heinrichs Markus , Frances S. Chen, Gregor Domes	2012	Social neuropeptides in the human brain: oxytocin and social behavior	Correlation /Treatment	Germany	*book*
HerpertzSC, I Schneider, C Schmahl, K Bertsch	2018	Neurobiological Mechanisms Mediating Emotion Dysregulation as Targets of Change in Borderline Personality Disorder	Treatment	Germany	Psychopathology
Jain, S	2014	Journal Watch review of Oxytocin and reduction of social threat hypersensitivity in women with borderline personality disorder.	Treatment	USA	Journal waTch review
Jawad MY , B Ahmad, AM Hashmi	2021	Role of oxytocin in the pathogenesis and modulation of borderline personality disorder: A review	Correlation	Pakistan	Cureus
Jobst Andrea , Frank Padberg, Maria-Christine Mauer, Tanja Daltrozzo, Christine Bauriedl-Schmidt, Lena Sabass, Nina Sarubin, Peter Falkai, Babette Renneberg, Peter Zill, Manuela Gander, Anna Buchheim	2016	Lower Oxytocin Plasma Levels in Borderline Patients with Unresolved Attachment Representations	Correlation	Germany	Frontiers in Human Neuroscience
Jobst Andrea ; Anna Albert; Christine Bauriedl-Schmidt; Maria Christine Mauer; Babette Renneberg; Anna Buchheim; Lena Sabass; Peter Falkai; Peter Zill; Frank Padberg	2014	Social exclusion leads to divergent changes of oxytocin levels in borderline patients and healthy subjects	Correlation	Germany	*Psychotherapy and psychosomatics*
Juraś-Darowny, D Strzelecki, M Talarowska	2023	Borderline personality - from psychoanalysis to epigenetics. Biological basis of attachment	Correlation	Poland	Psychiatria Polska
Kartal F, K Uğur, B Mete, ME Demirkol, L Tamam	2022	The Relationship Between the Oxytocin Level and Rejection Sensitivity, Childhood Traumas, and Attachment Styles in Borderline Personality Disorder	Correlation	Turkey	Psychiatry Investigation
Kirsch P	2015	Oxytocin in the socioemotional brain: implications for psychiatric disorders	Correlation /Treatment	Germany	Dialogues in clinical neuroscience
Kluczniok Dorothea , Katja Dittrich, Catherine Hindi Attar, Katja Bödeker, Maria Roth, Charlotte Jaite, Sibylle Winter, Sabine C. Herpertz, Stefan Röpke, Christine Heim & Felix Bermpohl	2019	Oxytocin and maltreatment potential. Influence of maternal depression, borderline personality disorder and experience of early childhood maltreatment	Correlation	Germany	*Der Nervenarzt*
Kohlhoff J , S Cibralic, DJ Hawes, V Eapen	2022	Oxytocin receptor gene (OXTR) polymorphisms and social, emotional and behavioral functioning in children and adolescents: A systematic narrative review	Correlation	Australia	Neuroscience & Biobehavioral Reviews
Leppanen J , KW Ng, YR Kim, K Tchanturia, J Treasure	2018	Meta-analytic review of the effects of a single dose of intranasal oxytocin on threat processing in humans	Tretment	Singapore/Sud Korea/UK/Georgia	Journal of Affective Disorders
Leppanen, J., Ng, K. W., Tchanturia, K., & Treasure, J.	2017	Meta-analysis of the effects of intranasal oxytocin on interpretation and expression of emotions	Treatment	UK	*Neuroscience & Biobehavioral Reviews*
Lischke A , SC Herpertz, C Berger, G Domes, M Gamer	2017	Divergent effects of oxytocin on (para-)limbic reactivity to emotional and neutral scenes in females with and without borderline personality disorder	Treatment	Germany	Social cognitive and affective neuroscience
Mancke F, SC Herpertz, K Bertsch - **Personality Disorders**	2015	Aggression in borderline personality disorder: A multidimensional model	Correlation	Germany	Personality Disorders: Theory, Research, and Treatment
Manuel C. D. Silva	2020	Oxytocin as a potential adjuvant treatment in borderline personality disorder – a review	Treatment	Spain	*European Neuropsychopharmacology*
Maoz H , A Grossman-Giron, O Sedoff, U Nitzan, H Kashua, M Yarmishin, O Arad, DT Bitan	2024	Intranasal oxytocin as an adjunct treatment among patients with severe major depression with and without comorbid borderline personality disorder	Treatment	Israel	Journal of affective disorder
Meyer-Lindenber, A., Domes, G., Kirsch, P., & Heinrichs, M.	2011	Oxytocin and vasopressin in the human brain: social neuropeptides for translational medicine	Correlation /Treatment	Germany	*Nature Reviews Neuroscience*
Mielke Emilia L , Julian Koenig, Sabine C Herpertz, Sylvia Steinmann, Corinne Neukel, Pelin Kilavuz, Patrice van der Venne, Katja Bertsch, Michael Kaess	2023	Adverse childhood experiences mediate the negative association between borderline personality disorder symptoms and plasma oxytocin	Correlation	Germany	Progress in neuro-psychopharmacology and biological psychiatry
Patin, A., & Hurlemann, R.	2015	Social cognition	Correlation	Germany	*Cognitive enhancement*
Peled-Avron L , A Abu-Akel, S Shamay-Tsoory	2020	Exogenous effects of oxytocin in five psychiatric disorders: a systematic review, meta-analyses and a personalized approach through the lens of the social salience hypothesis	Correlation /Treatment	Israel	Neuroscience & Biobehavioral Reviews
Perez-Rodriguez M.M., Yuan Q., Zhou Z., Hodgkinson C.A., Bevilacqua L., Ripoll L., Goodman M., Koenigsberg H.W., Shen P.-H., Goldman D., Siever L., New A.S.	2013	Oxytocin genotype may modulate reactivity to the environment in borderline personality disorder	Correlation	USA	*Neuropsycopharmacology*
Perez-Rodriguez, M. D. L. M.	2014	Neuropeptides and BDNF and emotion dysregulation in borderline personality disorder	Correlation	USA	*Biological Psychiatry*
Perez-Rodriguez, M. M.	2014	Converging multimodal evidence of social cognitive abnormalities in borderline and schizotypal personality disorders: Circuits, modulators and mechanisms	Correlation /Treatment	Usa	*Neuropsychopharmacology*
Perez-Rodriguez, M. M., Bulbena-Cabré, A., Nia, A. B., Zipursky, G., Goodman, M., & New, A. S	2018	The Neurobiology of Borderline Personality Disorder	Correlation	Spain	*Psychiatric Clinics*
Plett O , V Flasbeck, M Brüne	2023	Effects of human and animal-assisted skills training on oxytocin und cortisol levels in patients with borderline personality disorder	Treatment	Germany	Journal of Psychiatric Research
Ramseyer, F., Ebert, A., Roser, P., Edel, M. A., Tschacher, W., & Brüne, M.	2020	Exploring nonverbal synchrony in borderline personality disorder: A double-blind placebo-controlled study using oxytocin	Treatment	Switzerland	*British Journal of Clinical Psychology*
Ripoll LH	2012	Clinical psychopharmacology of borderline personality disorder: An update on the available evidence in light of the Diagnostic and Statistical Manual of Mental Disorders-5	Correlation	USA	Current Opinion in Psychiatry
Ripoll, L. H.	2013	Psychopharmacologic treatment of borderline personality disorder	Treatment	USA	*Dialogues in clinical neuroscience*
Saeed, S. A., & Kallis, A. C.	2021	Borderline personality disorder: 6 studies of biological interventions	Treatment	USA	*Current Psychiatry*
Schmitz M , LE Müller, A Schulz, N Kleindienst, SC Herpertz, K Bertsch	2020	Heart and brain: Cortical representation of cardiac signals is disturbed in borderline personality disorder, but unaffected by oxytocin administration	Treatment	Germany	Journal of Affective Disorders
Schneider I , S Boll, I Volman, K Roelofs, A Spohn, SC Herpertz, K Bertsch	2020	Oxytocin Normalizes Approach–Avoidance Behavior in Women With Borderline Personality Disorder	Treatment	Germany/UK/Netherlands	Frontiers in Psychiatry
Servan A , J Brunelin, E Poulet	2018	The effects of oxytocin on social cognition in borderline personality disorder	Treatment	France	L'encephale
Siever, L. J., Hodgkinson, C. A., Weinstein, S., Shen, P. H., New, A. S., & Goldman, D.	2010	Opioids and oxytocin: Genotypes and phenotypes in BPD	Correlation	USA	*Biological Psychiatry*
Simeon D , J Bartz, H Hamilton, S Crystal, A Braun, S Ketay, E Hollander	2011	Oxytocin administration attenuates stress reactivity in borderline personality disorder: a pilot study	Treatment	USA	Psychoneuroendocrinology
Stanley B , LJ Siever	2010	The interpersonal dimension of borderline personality disorder: toward a neuropeptide model	Correlation	USA	American Journal of Psychiatry
Stevens, F. L., Wiesman, O., Feldman, R., Hurley, R. A., & Taber, K. H.	2013	Oxytocin and behavior: Evidence for effects in the brain	Correlation /Treatment	USA	*The Journal of neuropsychiatry and clinical neurosciences*
Stoffers JM , K Lieb	2015	Pharmacotherapy for borderline personality disorder--current evidence and recent trends	Treatment	Germany	Current psychiatry reports
Vancova Z	2021	Potential therapeutic possibility of oxytocin for borderline personality disorder	Treatment	Slovakia	Psychiatric Annals
Zhang M , N Liu, H Chen, N Zhang	2020	Oxytocin receptor gene, childhood maltreatment and borderline personality disorder features among male inmates in China	Correlation	China	BMC psychiatry

Most of the manuscripts were published in the last 15 years, except for one article released in 2006.

#### 3.1.1 A psychoanalytic perspective

Bidirectional influences between maternal and infant OXT systems begin when the newborn is about 3 months old, enhancing both maternal care, infant social and emotional development and are essential to the development of attachment. Furthermore, the quality of parental care (warmth and availability) controls and modulates the development of a child's physiological and psychological infrastructures, as well as their gene expression. BPD is a disease characterized by difficulties and in attachment, a possible disruption in neuropeptides that regulate that system, such as oxytocin, are plausible and under investigation (5).

#### 3.1.2 Different attachment styles and oxytocin

Juraś-Darowny et al. found that oxytocin plasma levels did not differentiate by attachment style in healthy individuals, while it is different in BPD patients. OXT plasma levels in the anxious-avoidant style were significantly higher than in the anxious-preoccupied style (5, 6). Furthermore, as the majority of BPD patients exhibit unresolved (disorganized) attachment representations, this subset of patients expressed significantly lower baseline OXT plasma levels compared to BPD patients with organized attachment (7).

Interestingly, in borderline patients, especially those with unresolved attachments, oxytocin levels were shown to decrease significantly after separation and not increase normally as in controls. This evidence highlights the role of oxytocin in higher rejection susceptibility due to greater efforts to avoid denial which is an epitome of BPD (7, 8).

#### 3.1.3 An implication for compassion

These results have significant ramifications for therapeutic environments as well. The attachment system is stimulated in therapy and may elicit emotional memories. Research on the function of resistance to these emotions and relationships is crucial in the treatment of BPD patients.

It became evident, during the clinical development of compassion-focused therapy, that many clients had significant avoidance of self-compassion (with feelings of anger and anxiety, feeling alone and yearning for closeness, fear) and resistance to be open and responsive to compassion from others (Fear Of Compassion-FOC). Compassion is associated with caring motivations, which are neurochemically connected to the action of oxytocin.

Usually, for BPD patients, providing care for others was characterized by a lack of availability, neglect, emotional insensitivity, or even abuse and harm.

Ebert et al. discovered a negative correlation between OXT levels and FOC in individuals from the BPD group. In a recent study they showed that, in comparison to the control group, BPD patients exhibited noticeably greater fears and resistances to all forms of compassion. Additionally, BPD patients remembered their parents' actions from their childhood less favorably. Lack of emotional warmth may have an impact on a child's OXT system that is comparable to a mild trauma. This may suggest that this ìpopulation of patients would benefit from specialized focused treatment in this field (6).

#### 3.1.4 OXT and mother-child interactions

Being a mother implies behavioral, physiological, and neurological changes that are crucial for successful mother-child interactions and sensitive caregiving OXT plays an essential role in those changes. Herpertz et al. state thatAfter interacting with their own child, mothers with BPD exhibited altered oxytocin and cortisol reactivity, with a decrease in oxytocin and an unchanged cortisol level; on the contrary, mothers without BPD displayed stable oxytocin levels and a decrease in cortisol after mother-child interaction (9).

According to the theoretical implications of this model on parent-child relationships, low parental oxytocin levels are linked to a decreased sense of reward during interactions with their offspring with poor importance given to the child's requests. These factors reduce parental sensitivity and may contribute to insecure attachment and poor parent-child bonding (9–11).

Furthermore, a child's oxytocin levels may be lowered as a result of the mother's decreased OXT levels (9, 10).

On the other hand, Kluczniok et al. tested OXT as a potential mediator for the association between maternal experience of early childhood maltreatment and potential abuse perpetrated against their own child in BPD. They found that early childhood maltreatment is associated with reduced plasma OXT in mothers with mental distress due to several factors (including BPD). Notwithstanding, OXT low level did not mediate the association with abusing of their own child (12).

### 3.2 A Biopsychosocial perspective

Finding specific factors linked to borderline personality disorder (BPD) and low plasma levels of oxytocin in BPD patients may help in the identification of those patients who would benefit from therapies with oxytocin (13–15).

#### 3.2.1 OXT and early life stressful event

Unfavorable early life and childhood experiences, such as persistent rejection, maltreatment and abandonment are important environmental elements that may influence the oxytocin system (11). Early stress can modify the activity of the oxytocin receptors and interfere with both its binding properties and the neuropeptide system's development (16).

BPD is associated with lower levels of oxytocin as well as decreased expression of the oxytocin receptor (OXTR), indicating the involvement of both oxytocin and its receptor in this disorder (17).

As a possible consequence, brain altered neurodevelopment in BPD patients with a history of trauma and early life stress may be related to an impairment in the oxytocin system. The amygdala, which is responsible for the emotional regulation which is lacking in BPD patients, may suffer from the interaction between the OXTR gene and childhood maltreatment.

According to research by Bartz et al., severe early stress and maltreatment can affect the oxytocin–vasopressin stress–response system, which, in turn, can alter brain development and cause a variety of disorders, such as BPD and Post Traumatic Stress Disorder (PTSD). Prolonged early stress modifies the release of corticotropin, which then modifies the binding of the oxytocin and vasopressin receptors. Some authors have also proposed that gonadal steroids and oxytocin may play a role in the way that stress in early life influences affiliative behaviors in adults (18–21).

#### 3.2.2 The interaction between gene and environment in BPD

The interaction between the gene and environment (G × E) may play a role in the development of BPD (22–24). It is important to view OXTR from the perspective of "differential susceptibility" since a reduced protein expression due to different genotypes may be a biological mediator of some aspects of the psychogenesis and psychopathology of BPD. OXTR is on chromosome 3p25.3 of the human genome. A growing number of researches, including reports of both direct effects and gene-environment interactions, points to the possibility that single nucleotide polymorphisms (SNPs) of the OXTR gene might be linked to individual variations in social and emotional abilities as well as to the genesis of psychopathology (25).

The OXTR's rs53576, a SNP in the third of four introns, is the most thoroughly studied single-nucleotide polymorphism (SNP) (26, 27). Since the frequency of rs53576 varies according to ethnicity, genetic susceptibility may also differ between racial groups. Another explanation for these contradictory effects of genetic susceptibility could be gender differences, which has been extensively documented in the literature.

Moreover, according to a recent study, females with the A allele were more likely to experience maltreatment as children. This could indicate that different genders have distinct patterns of interaction between the OXTR gene and childhood maltreatment (22).

Furthermore, Kohlhoff and colleagues found that BPD-OXTR rs53576 moderate the interaction between child maltreatment and BPD traits, with varying effects in each genders, but it did not directly predict BPD traits.

In particular, girls who had suffered abuse and belonged to the AG-AA group (presence of minor allele A) showed more severe BPD traits compared to girls with the GG genotype who had not been abused.

Boys who were maltreated and had the GG genotype exhibited more BPD traits than boys who were not, with a specific gender-oriented model. On the contrary, there was no difference in the degree of BPD traits between boys with the AG-AA genotype and those who were not abused (2).

The OXTR gene is thought to contribute disproportionately to disturbed relatedness. When early parental warmth is present, AA/AG genotypes are less likely to develop BPD; however, when early childhood abuse occurs, they are more likely to develop psychopathology (28, 29).

Studies examining polymorphisms in OXTR revealed that multiple variants of the gene are linked to empathic concern for other people as well. The goal of Jawad et al. study was to investigate how oxytocin receptor gene variants interact with childhood trauma, detected with the Childhood Trauma Questionnaire (CTQ), and affect a person's capacity for BPD empathy in case of painful experiences. They discovered that low scores on the CTQ were linked to less empathy for psychological pain, while childhood maltreatment in A-allele carriers was associated with higher empathy for psychological pain. According to this evidence, people who have at least one A-allele are more vulnerable to environmental variation, which may have a secondary role in the onset of emotional instability (26, 28).

#### 3.2.3 Phenomenology of borderline personality disorder and oxytocin

In current literature, the three main psychopathological domains of borderline personality disorder (BPD) are Behavioral dysregulations—impulsivity (suicidal thoughts and behaviors, and self-mutilation), affective dysregulation (which includes inappropriate anger and attempts to prevent abandonment) and disturbed relatedness (unstable relationships, identity disorders, and a persistent sense of emptiness) (2). A reduced protein expression may be a biological mediator of some aspects of the psychogenesis and psychopathology of BPD (17).

#### 3.2.4 The “threat hypersensitivity” as the core of BPD symptoms

The "threat hypersensitivity", thought to be a common trait in people who experienced interpersonal maltreatment in their early childhood, is closely linked to the three domains of BPD psychopathology.

Furthermore, “threat hypersensitivity” is linked to amygdala hyperactivity, which is the salience network activated in response to threatening social stimuli. This phenomenon is known as enhanced emotional dysregulation or "bottom up emotion generation."

According to the social salience theory, OXT makes people more sensitive to social cues and intrapersonal and interpersonal variables determining if a person to react positively or negatively.

In particular, the abnormal stress response and the top-down and bottom-up dysregulation of emotional systems that characterize Borderline Personality Disorder might be influenced by oxytocin deficiencies.

Due to inadequate prefrontal cortex modulation, low oxytocin action on the limbic system would cause an abnormally high amygdala activation, leading to interpersonal hypersensitivity, emotional dysregulation, and impulsive behavior (17).

These results are also consistent with a process of down-regulation of the oxytocin system in BPD patients as a reaction to early adverse experiences linked to disordered attachment styles, as previously reported by Jobst et al. (7)

Bomann et al. described positive correlations between plasma OXT levels and exposure to uncontrollable noise, various social stressors, and attachment anxiety, suggesting that oxytocin has a calming and stress-relieving effect. This suggested relationship between serum OXT and BPD symptomatology is thus demonstrated by this evidence (2, 30).

#### 3.2.5 Emotional and behavioral dysregulations

Reduced basal levels of oxytocin cause the amygdala to become more activated in BPD patients, impairing their ability to comprehend social cues and resulting in aberrant behaviors and emotional dysregulation (31). This theory is further supported by the discovery of a strong negative correlation between psychological self-reported stress, anxiety, anger traits and OXT (32).

Byrd et al. described an intriguing link between early threat experiences, oxytocin receptor genetic assets in BPD females and traumatic childhood exposure. Women who carry at least one copy of the rs53576 gene and A allele, showed increased levels of emotion dysregulation in adolescence. This evidence was predictive of psychopathology in general, including BPD traits and diagnosis, in all women (20, 25).

#### 3.2.6 Aggressive behaviors

Similarly, reactive aggression has been negatively related to the oxytocin system (33). Trait aggressiveness and self-reported aggressiveness were inversely correlated with oxytocin plasma concentrations in BPD patients (34), confirming the possibility that abnormal impulsive-aggressive behavior, which is typical of BPD patients, is strictly related to oxytocin system dysfunction (15).

Considering possible genetic implications, De Las Mercedes Perez-Rodriguez et al. discovered a risk linked to haplotype CT of the oxytocin gene (rs877172 C rs3761248 T), which is associated with increased aggression and anxious attachment (31). Furthermore, Siever et al. found a link between four OXT SNPs and inappropriately high levels of anger in BPD patients (35).

A recent study (36) attested a correlation between OXT SNPs and the Personal Distress subscale of the Interpersonal Reactivity Index (a measure of dispositional empathy) in adults with personality disorders, as well as irritable aggressive anger dysfunction. According to this data, the oxytocin system may modulate reactivity to the environment in BPD.

#### 3.2.7 Suicidal and self-harming behaviors

BPD is frequently associated with emotional,behavioral dysregulation andimpulsive aggression as welland suicidal and self-harming behaviors in response to perceived rejection and loss,.

In BPD, there is a clear correlation between self-harm and reduced pain sensitivity. Oxytocin modulates pain by interacting with the opiate and cannabinoid systems. According to literature, oxytocin-positive parvocellular neurons in the hypothalamus project directly to the brainstem, where they may work with modulatory signals from peripherally released oxytocin to reduce both physical and psychological pain (37).

#### 3.2.8 Interpersonal dysfunction and social cognition

Patients with borderline personality disorder may have relational difficulties, which have been explained by social deficits and emotional dysregulation.

In order to examine the acknowledged role of OXT in attachment, especially in romantic partner bonding, Bomann et al. investigated potential associations between serum OXT and marital status in BPD.

The mean serum OXT level of patients in romantic relationships was 599 pg/ml, while the mean level of single patients was 374 pg/ml. This disparity represented a significant difference between the two groups.

These findings could reinforce the evidence that OXT is a crucial mediator of romantic attachment. However, since BPD patients frequently suffer significant emotional distress and intense fear of being abandoned, it is plausible that their elevated OXT serum levels reflect a higher level of stress than among singles. Higher OXT plasma levels, in this study, have been linked to self-reports of relationship distress and relationship anxiety, supporting this theory (30).

Affective dysregulation alone cannot fully account for low interpersonal functioning while social cognition impairment may also be a factor. These deficits in social cognition are supposed to be associated with unfavorable early life experiences and are primarily mediated by the oxytocin system, which has been demonstrated to be controlled by epigenetic modifications (22, 34). In response to a scenario of social exclusion, which is a powerful negative bonding stimulus in BPD, Jobs et al. found that female BPD patients had lower OXT plasma levels than healthy individuals (7, 32).

Jobst et al. examined the plasma levels of oxytocin in both borderline patients and healthy controls (HC) following Cyberball, which is based on social exclusion and causes social pain in people. They showed that there was no difference in oxytocin levels despite attachment representations. Similar OXT variations following social exclusion were detected in BPD patients with organized attachment as well as in those with unresolved attachment representations. However, OXT plasma levels were lower in the latter group (7).

When OXT plasma levels were directly compared between the BPD and HC groups following social exclusion, a significant difference was observed. BPD patients' OXT plasma levels tended to decrease, while HC patients tended to increase. OXT plasma levels were consistently lower in BPD patients than in HC.

Such results indicate that BPD and HC have distinct emotional reactions to social exclusion. In this particular situation, OXT release may encourage prosocial behavior and lessen social discomfort. This OXT response to social exclusion may be compromised in BPD patients, which may be related to their overall difficulty in mending damaged cooperation skills (7).

Interestingly, the OXT system grows as a result of experiences that occur during sensitive developmental periods, and it might be particularly affected by traumatic childhood experiences. Epigenetic modifications of genes implicated in OXT signaling may be involved in the mechanisms mediating the long-term influence of early adverse experiences on socio-behavioral outcomes.

On the one hand, "Low OXT state" could refer to a protective state where negative emotions are less perceived and social pain might be easier tolerated. In this special context, early childhood maltreatment and the formation of insecure attachment representations might be linked to the development of borderline personality disorder and may contribute to the symptoms of BPD behavior in adulthood (38).

### 3.3 Oxytocin as a treatment for BPD

The role of Oxytocin in the treatment of BPD is complex, ambivalent and still under investigation. Several studies have been conducted in the last years aiming to discover the therapeutic potential of this hormone for this specific disease.

Some studies demonstrated promising results for a possible treatment of some key symptoms of the BPD with Intranasally administration of Oxytocin.

#### 3.3.1 Social threat

Oxytocin have an impact on social threat avoidance and in social threat hypersensitivity in subject affected by BPD, As attested by Schneider et al. (39) oxytocin normalizes the threat-avoidance behavior in patients with BPD by enhancing reaction times in affect-incongruent (approach angry and avoid happy faces) conditions. The results of the study by Bertsch et al. (40) showed beneficial effects from administration of oxytocin stressing a decreasing social threat hypersensitivity and reducing eventually anger and aggression in patients with BPD. Similarly, according to the results of the study by Brüne et al. (38) oxytocin modifies the avoidant response to social threat in BPD patients, probably lowering stress level and inhibiting social withdrawal from distressing social stimuli.

#### 3.3.2 Stress reactivity

Patients diagnosed with borderline personality disorder often describe their lives as stressful and unpredictable. In the study by Simeon et al. (16) a single-dose OXT administration in a group of BPD patients attenuated dysphoric emotional response and lowered the cortisol response to stress. Lischke et al. (41) showed that oxytocin decreased amygdala and insula reactivity in BPD patients, suggesting that oxytocin may be capable of attenuating BPD patients’ stress and hypersensitivity for complex scenes, irrespective of their valence.

#### 3.3.3 Empathy and approach motivation

Dysfunction of empathy and related processes in patients with BPD has been widely suggested. Domes et al. (42) give evidence of a beneficial effect of a single dose of oxytocin on affective empathy and approach motivation in women with BPD adapting their level of social functioning to healthy controls with important clinical implications for the future treatment of the disease.

Although promising results that can lead to further investigations on oxytocin as a possible treatment for BPD, OXT didn’t show an impact on other elements of the disease.

#### 3.3.4 Nonverbal behavior

Nonverbal behavior consists of actions that can indicate an individual’s attitudes or feelings without speech. Nonverbal behavior can be apparent in facial expressions, gaze direction, interpersonal distance, posture and postural changes, and gestures. It serves several functions, including providing information to other people (if they can detect and understand the signals), regulating interactions among people, and revealing the degree of intimacy among them. Nonverbal behavior is often used synonymously with nonverbal communication, even though nonverbal actions are not always intended for, or understood by, other people.

Oxytocin seems to have a negative impact on nonverbal behavior, an important factor in interpersonal relationships.

Brüne et al. (43) analyzed the impact of OXT in modifying non-verbal signs in BPD patients and they didn’t find any positive change in their non-verbal communication after OXT administration. Instead, oxytocin acted in a pro-social way in clinically healthy subjects. Oxytocin administration seems also to worsen non-verbal synchrony in BPD patients, while OXT administration enhances non-verbal synchrony in mentally health subjects.

#### 3.3.5 Mentalizing

Mentalizing -the accurate understanding of mental states- is a domain belonging to social cognition. Two types of mentalizing errors have been described: Hypo-mentalizing errors are simplistic interpretations of social cues, likely due to deficits in social information processing. Hyper-mentalizing errors are distorted misinterpretations of social cues, likely due to hypersensitivity to social stimuli. As demonstrated by the results of Fisher et al. (44) and Ha et al. (45) OXT doesn’t positively affect the hyper-mentalizing phenomenon in BPD patients; instead OXT seems to worsen it due to intensifying.

#### 3.3.6 Trust and cooperation

Oxytocin, although it has been regarded colloquially as a prosocial hormone, has a trust-lowering effect in BPD, which was correlated with patients' history of childhood trauma (46). The study by Bartz et al. (18) highlights the effect on trust in BPD patients and agrees with previous studies that OXT does not uniformly facilitate trust and pro-social behavior in humans; in fact, OXT may impede trust and pro-social behavior depending on psychiatric diagnosis (e.g. BPD) and/or chronic interpersonal insecurities combined with situational factors that heighten those insecurities.

#### 3.3.7 Cardiac alterations in BPD

Heart rate variability (HRV) is the physiological phenomenon of variation in intervals between heartbeats. It is measured by the variation in the beat-to-beat interval. HRV is related to emotional arousal and alterations of HRV is related to many psychiatric disorders.

Reduced heart variability (HRV) is associated with self-regulatory deficit in BPD. Back et al. (47) investigated the possibility of modification of HRV in the resting state of BPD patients by oxytocin. Their results showed OXT did not have a significant effect in the modification of the HRV. Furthermore,oxytocin administration, according to the study by Schmitz et al. (48), doesn’t modify heartbeat-evoked potentials (HEPs) as a marker of the cortical representation of cardiac signals in BPD.

## 4 Discussion

This is, to our knowledge, the first extensive review about the role of oxytocin in borderline personality disorder and its possible contribution as a treatment.

The interplay between maternal and infant oxytocin (OXT) systems highlight their crucial role in attachment development (5, 9–11). The quality of parental care is impacted, as it modulates a child's physiological and psychological infrastructures, potentially influencing gene expression. In patients with Borderline Personality Disorder (BPD), disruptions in the oxytocin system are implicated in attachment difficulties and resistance to compassion.

The findings suggest that attachment styles might be linked to oxytocin plasma levels, particularly in BPD patients. Those with anxious-avoidant attachment styles exhibit higher oxytocin levels compared to those with anxious-preoccupied styles. Furthermore, individuals with unresolved attachment representations show lower baseline oxytocin levels. This supports the hypotheses that oxytocin may play a role in susceptibility to rejection and avoidance behaviors, an epitome of BPD. Furthermore, early life stress, including maltreatment, can influence oxytocin levels and its receptor expression, potentially contributing to BPD development.

Therapeutically, the comprehension of the role of oxytocin in attachment and compassion could support interventions like Compassion-Focused Therapy (CFT). Addressing oxytocin-related deficits in BPD patients may be crucial for improving their responsiveness to compassionate care (5).

Moreover, oxytocin's influence on mother-child relationship underscores its importance in sensitive caregiving. BPD mothers show altered oxytocin and cortisol reactivity after interacting with their children, which may influence parent-child bonding.

Early life stress and genetic factors influence oxytocin levels and receptor expression, impacting BPD development (17, 22, 24–26, 29). Research suggests that childhood maltreatment may lead to oxytocin system alterations, contributing to BPD symptoms.

Genetic variations in the oxytocin receptor gene (OXTR) are linked to social and emotional skills, as well as psychopathology. Gender differences and gene-environment synergy further complicate the relationship between OXTR polymorphisms, childhood maltreatment, and BPD traits. The "threat hypersensitivity" core of BPD symptoms is associated with oxytocin deficiencies, affecting emotional regulation and social cognition. Research indicates correlations between oxytocin levels and emotional dysregulation, aggression, and interpersonal dysfunction in BPD patients.

Studies highlight oxytocin's modulation of stress reactivity, empathy, and approach motivation in BPD patients. However, oxytocin administration doesn't improve all BPD symptoms. It may exacerbate non-verbal communication deficits, hyper-mentalizing, and trust issues in BPD patients, potentially due to their history of childhood trauma.

The therapeutic potential of oxytocin for BPD is complex and varied. While some studies suggest benefits in reducing emotional dysregulation, aggression, and stress reactivity, others indicate no significant effects or even negative impacts on trust, empathy, and non-verbal behavior.

## 5 Conclusion

In conclusion, this comprehensive overview provides valuable insights into the intricate role of oxytocin in borderline personality disorder (BPD) and its potential as a treatment option.

Attachment styles appear to correlate with oxytocin plasma levels, particularly in BPD patients, suggesting a potential role for oxytocin in susceptibility to rejection and avoidance behaviors typical of this disorder. Moreover, early life stress, including maltreatment, can influence oxytocin levels and receptor expression, contributing to BPD development. Genetic polymorphisms in the oxytocin receptor gene (OXTR) make the relationship between oxytocin, childhood maltreatment, and BPD traits more complicated, suggesting a potential gene-environment interaction in BPD development.

From a therapeutic point of view, while studies indicate correlations between oxytocin levels and emotional dysregulation, aggression, and interpersonal dysfunction in BPD patients, the therapeutic promise of oxytocin for BPD remains challenging and varied. While some studies suggest benefits in reducing emotional dysregulation, aggression, and stress reactivity, others show no significant effects or even negative impacts on trust, empathy, and non-verbal behavior.

Overall, this review highlights the multifaceted nature of oxytocin's involvement in BPD and emphasizes the need for further research to clarify its therapeutic potential and mechanisms of action in treating BPD symptoms.

## Author contributions

EdG: Conceptualization, Data curation, Formal analysis, Funding acquisition, Investigation, Methodology, Project administration, Resources, Software, Supervision, Validation, Visualization, Writing – original draft, Writing – review & editing. EA: Data curation, Formal analysis, Investigation, Writing – original draft. JS: Funding acquisition, Visualization, Writing – review & editing. AA: Data curation, Formal analysis, Investigation, Writing – original draft. MC: Funding acquisition, Supervision, Writing – review & editing.
